# A simple Bayesian estimate of direct RNAi gene regulation events from differential gene expression profiles

**DOI:** 10.1186/1471-2164-12-250

**Published:** 2011-05-20

**Authors:** Paul A Wilson, Mathew Plucinski

**Affiliations:** 1 Computational Biology, GlaxoSmithKline Medicine Research Centre, Gunnels Wood Road, Stevenage, SG1 2NY, UK; 2 Department of Applied Mathematics and Theoretical Physics, University of Cambridge, Wilberforce Road, Cambridge CB3 0WA, UK

## Abstract

**Background:**

Microarrays are commonly used to investigate both the therapeutic potential and functional effects of RNA interfering (RNAi) oligonucleotides such as microRNA (miRNA) and small interfering RNA (siRNA). However, the resulting datasets are often challenging to interpret as they include extensive information relating to both indirect transcription effects and off-target interference events.

**Method:**

In an attempt to refine the utility of microarray expression data when evaluating the direct transcriptional affects of an RNAi agent we have developed SBSE (Simple Bayesian Seed Estimate). The key assumption implemented in SBSE is that both direct regulation of transcription by miRNA, and siRNA off-target interference, can be estimated using the differential distribution of an RNAi sequence (seed) motif in a ranked 3' untranslated region (3' UTR) sequence repository. SBSE uses common microarray summary statistics (*i.e*. fold change) and a simple Bayesian analysis to estimate how the RNAi agent dictated the observed differential expression profile. On completion a trace of the estimate and the location of the optimal partitioning of the dataset are plotted within a simple graphical representation of the 3'UTR landscape. The combined estimates define the differential distribution of the query motif within the dataset and by inference are used to quantify the magnitude of the direct RNAi transcription effect.

**Results:**

SBSE has been evaluated using five diverse human RNAi microarray focused investigations. In each instance SBSE unambiguously identified the most likely location of the direct RNAi effects for each of the differential gene expression profiles.

**Conclusion:**

These analyses indicate that miRNA with conserved seed regions may share minimal biological activity and that SBSE can be used to differentiate siRNAs of similar efficacy but with different off-target signalling potential.

## Background

RNA interference (RNAi) is an evolutionary conserved mechanism that has been observed as a key component of many cellular development and differentiation processes [1,2]. Two intensely studied effectors of RNAi are the microRNAs (miRNA) and the small interfering or silencing RNAs (siRNA). Both entities are processed via the Dicer biogenesis pathway and their inherent transcriptional regulatory processes overlap in many aspects [3-5]. It has been estimated that there are approximately 900 human miRNA most of which are poorly characterised with regard to both their biological targets and cellular functionality [6,7]. However, a number of human miRNAs are reported to have causative roles in human disease and it is predicted that many more are intrinsically involved in both the generation and maintenance of other pathological conditions [10,11]. A better understanding how miRNAs evoke a disease condition is of immense interest and is the focus of a huge research effort. In contrast, synthetic siRNAs are exogenous entities that also hold huge potential as human therapeutics as they have the ability to specifically repress transcription of disease-causing genes [12,13].

It is generally accepted that miRNA regulate gene expression at the post-transcriptional level via translation arrest and mRNA cleavage in association with the RNA-induced silencing complex (RISC) [3,14]. The regulatory mechanism is reliant on partial complementarity between the nucleotides of the miRNA and the 3'UTR (untranslated region) of target mRNAs. Of critical importance in the targeting mechanism is a "seed" region at the 5' of the miRNA spanning residue positions 2-8 [15,16]. In contrast, synthetic siRNA specificity is dependent on complete complementarity between the siRNA sequence and the target mRNA [12,17]. However, it has been observed that many siRNA also exhibit "off-target" effects (*i.e*. repress non-target mRNA). Studies indicate that these effects can be either 'generic' (*e.g*. trigger the innate immune response) or sequence-specified miRNA-like events between nucleotides at the 5' end of the siRNA and the 3' UTR of non- target mRNA [20-23].

Microarray technologies provide an unbiased snap-shot of the cellular transcriptional activity, and they are often employed to investigate both the functional and biological characteristics of miRNA and siRNA in various cell-lines, under varying physiological conditions [24,25]. However, it remains a challenge to identify those differentially regulated transcripts that are direct targets of the transfected miRNA or siRNA (*i.e*. sequence-specified) from those that are 'indirect' events (*e.g*. a signalling event as a consequence of perturbing the cellular network). Often a small number of differentially regulated transcripts are investigated in further detail (*e.g*. via real-time quantitative reverse transcription), but such approaches are time consuming, labour intensive and make minimal use of the dataset as a whole.

To address this issue a variety of computational approaches have been developed. For example, a number of algorithms have been used to computationally predict miRNA targets [26,27], and these predicted mRNA targets are in turn 'mapped' to the list of differentially regulated transcripts. However, it has been observed that there is little agreement between current miRNA prediction algorithms [28,26], which reduces confidence in this approach. The HOCTAR method [25] extends on this approach by utilising inverse correlations between 178 intragenic human miRNA that are present on the Affymetrix HG-U133 microarray and predicted miRNA gene targets down-regulated following miRNA transfection. As with the former approach described, HOCTAR is reliant on low-confidence target predictions and has limited application beyond the HG-U133 platform. The Sylamer algorithm [29] offers a significant alternative to prediction based methods as it has general applicability (*i.e*. it is not platform dependent and can be used with both miRNA and siRNA derived datasets) and is independent of third party prediction algorithms. Sylamer estimates for enrichment of an RNAi motif given a list of differentially expressed gene identifiers and reports any RNAi induced bias within a composite plot of the hypergeometric p-values estimated for all other nucleotide "words" of the same length (as the seed query sequence). However, the over-representation bias of the RNAi 'seed' sequence is often reported as a broad peak that encompass much of the dataset making it difficult to ascertain a suitable "cut-off" threshold for validation efforts. On other occasions no significant over-representation is reported despite differential expression data suggesting a significant RNAi induced response (See Additional File 1 for comparative plot examples). These combined observations suggest that the sensitivity of the method could be improved.

In an effort to improve on these current limitations we have engineered an alternative and possibly more sensitive 'seed' estimation method that utilises a Bayesian likelihood approach to estimate the probability that a 'seed' motif is over-represented within a differentially expressed gene profile. Significant enrichment scores are interpreted as evidence of 'direct' RNAi and provide a relative estimate of the magnitude of such activity. SBSE has been evaluated using a number of diverse RNAi microarray datasets, several of which are reported here. Analysis of a miRNA time-study allowed us to visualise the transient nature of miRNA directed events and indicates that SBSE could be used to determine the optimum timing of a post-transfection investigation of the direct miRNA transcription effect. Furthermore, our analyses indicates that miRNA with conserved seed regions may share minimal RNAi activity, and that SBSE can be used to differentiate otherwise equivalent siRNAs via estimates of their respective unique miRNA-like off-target profiles.

## Results

**Figure 1:**
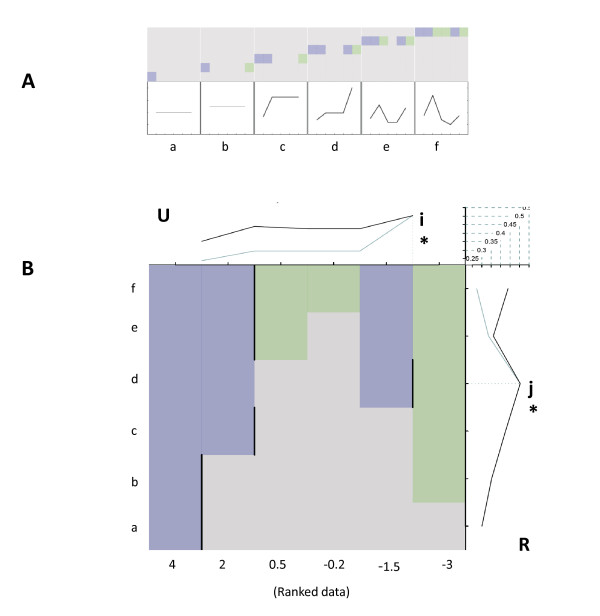
**A cartoon representation of the SBSE analysis procedure**. **Panel A: **The top row illustrates how each of the six observations (a-f), represented by coloured blocks, are sequentially evaluated by the algorithm. The bottom row represents the -log(p-value) associated with each hypothesis update. Column (a) represents the first evaluation, column (b) the second evaluation *etc*. Using the hypothetical data described in the main text, the algorithm first encounters a grey box (*i.e*. an up-regulated gene not containing a seed sequence match) and estimates that there is a one-half chance that the observed differential expression profile can be best explained by the differential distribution of the seed motif. Next, a green box (*i.e*. a down-regulated gene containing a seed sequence match) is encountered and the hypothesis is updated accordingly. The algorithm continues to update the hypothesis until all of the data has been processed and a p-value calculated for each subsequent observation. The estimates for the complete dataset are combined and summarised as illustrated in **Panel B: **Each of the six differentially expressed genes, sorted from most up-regulated to most down-regulated (*i.e*. left-to-right), are represented by the x-axes, with a green shaded column indicating the presence of the miRNA seed motif and grey shading indicating the absence of the seed motif. Each row (a-f) represents the hypotheses evaluated at each step of the analysis procedure as described for panel A. The black vertical lines in each row of the central section of the plot indicate the optimal division of the data at that juncture. The upper-most section (**U**) of the plot summarises the -log of the estimated p-values. The optimum partition of data is indicated by a faint vertical dashed blue line (i*) emerging from the point of the most significant p-value. The right-most section (**R**) of the plot also summarises the -log of the estimated p-values associated with each hypothesis update. The faint horizontal blue line (j*) indicates the most significant p-value and indicates those transcripts considered important in our estimate of i*. Both the uppermost and rightmost plots use the same scaled axes and may be used to best partition the data for further focussed analyses. In this theoretical expression profile, the most significant differential distribution of the miRNA seed motif is best estimated using data from the top four transcripts and, by inference, any direct miRNA effect restricted to the transcript represented by column six which is located to the right of i*, the largest enrichment score. Note that the order in which each observation is incorporated into the analysis is dictated by the absolute ranked vector and that for large and normally distributed datasets the main section of the summary plot will form a triangle as the algorithm processes the data from most to least dysregulated transcript.

How the SBSE algorithm was implemented is summarised in cartoon format (See Figures 1A and 1B) and outlines the analysis of a pseudo dataset. This approach was extended to process larger datasets such as that encountered when using the Human Genome U133A Plus 2.0 Affymetrix GeneChip^®^. The described microarray datasets were selected as representatives of the diversity of RNAi investigations that would most likely be encountered in a 'typical' RNAi analysis. The 3'UTR human sequences necessary for estimation of the query (seed) motif enrichment were parsed and repetitive nucleotide motifs masked (available as Additional File 2). All differentially expressed Affymetrix probe set identifiers, along with their associated fold change and p-value, were generated using standard microarray analysis methodology (See Methods) and accessed via tab-delimitated format (all datasets available as Additional File 3). Each of the differential transcript lists were used as the respective query inputs in the evaluation of SBSE as described.

### Case study 1

E-GEOD-6207 comprised 14 Affymetrix GeneChip^® ^Human Genome U133A Plus 2.0 cel files. In this study [7] hsa-miR-124 (*i.e*.UAAGGCACGCGGUGAAUGCC) was over expressed in HepG cells and RNA extracted at time points 0, 4, 8, 16, 24, 32, 72 and 120 hours post-transfection to identify gene transcripts down-regulated by hsa-miR-124 over expression. Our primary hypothesis regarding this dataset was that should hsa-miR-124 selectively down-regulate target transcripts via a seed-pairing directed mechanism then the nucleotide complement of the hsa-miR-124 seed sequence should be preferentially enriched in the down-regulated transcript population. Should our hypothesis prove correct, this approach could then be extended to further elucidate the degree of miRNA sequence conservation associated with hsa-miR-124 RNAi by iteratively querying with 'overlapping' variations of the hsa-miR-124 seed region.

**Figure 2:**
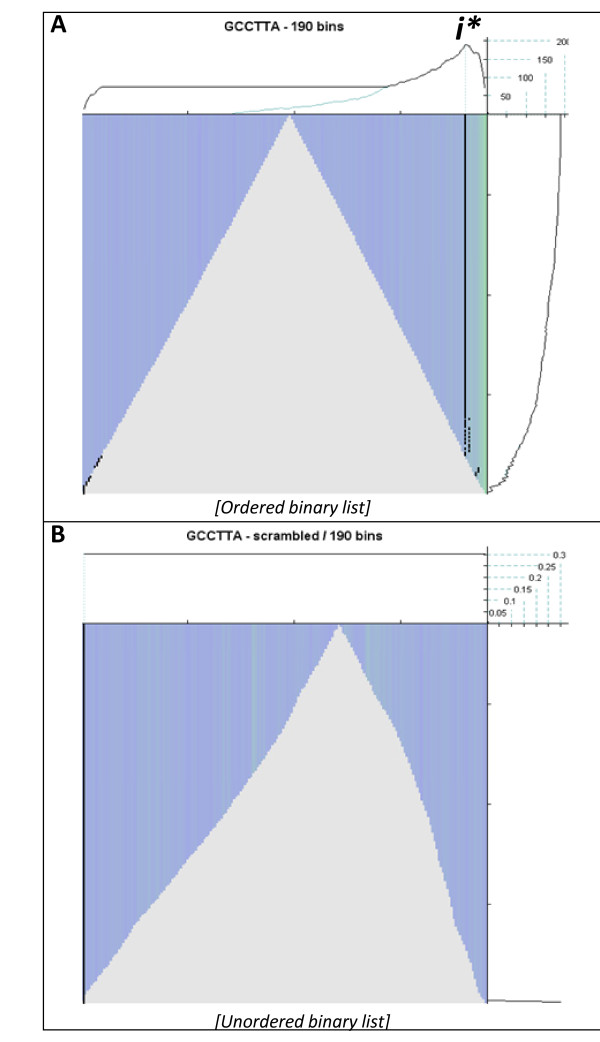
**Plots summarising the estimated location of the direct target transcripts of hsa-miR-124 16 hours post-transfection**. For each plot the x-axis represents the differential expression dataset organised by fold change and is ranked from most up-regulated (left) to most down-regulated (right). The central body of the plot represents how the algorithm traversed the dataset with green vertical lines highlighting a perfect match between the target 3' UTR and the RNAi seed sequence, and blue vertical lines the absence of perfect match. The characteristic triangle emphasises the broadly 'normal' distribution of the dataset (*i.e*. no overall bias towards up- or down-regulation). The analysis process is directed by the absolute ranking vector (see methods for further detail) and each data point is evaluated sequentially - from the outermost, and most dysregulated transcripts toward the central unaffected transcripts. The prominent black line indicates the location of the estimated optimal partitioning of the dataset with regard to the enrichment of (putative) direct RNAi targets. The uppermost plots of panels **A **and **B **trace the enrichment score and attempts to locate the most significant partitioning of the data throughout the analysis procedure. Note that the maximum enrichment score is indicated by "i*". The rightmost plots of panels **A **and **B **also describe the enrichment score, that is, but in this context summarise how the estimate of the enrichment score fluctuates as sequential data is processed. See methods section for further details. **(Panel A) **The data input was the differential expression profile as determined by the LIMMA statistical model. Note that SBSE estimates that the most significant grouping of hsa-miR-124 direct transcript targets are located to the right of the vertical line and are included amongst approximately 15% of the most down-regulated transcripts. **(Panel B) **The equivalent analysis to that described for panel A, but with the expression profile input shuffled. Note that the previous hsa-miR-124 signature has been abrogated and that there is now an absence of a significant estimate or partitioning of the data.

First the ranked, differential expression profile of each respective time point, relative to the 0 hour array, was queried for enrichment of a "GCCTTA" motif (*i.e*. the nucleotide complement of the hsa-miR-124 seed region). The resulting SBSE summary plots are illustrated with the analysis of the 16 hour profile (See Figure 2A). Our analysis indicated significant enrichment of the query motif in the most down-regulated transcripts and generated a maximum enrichment score of 190 (indicated by i*). Significant enrichment scores were observed with all analyses of later time point expression profiles (further details below). To rule out the possibility that these were random observations inherent in a large population each dataset was shuffled (*i.e*. by randomly sorting the unique transcript identifiers relative to the statistical descriptors) and each query repeated. In every instance this simple shuffling of the data completely abrogated detection of the enrichment signal, supporting the SBSE score as a robust estimate of seed enrichment in a differentially expressed dataset (Figure 2B).

**Figure 3:**
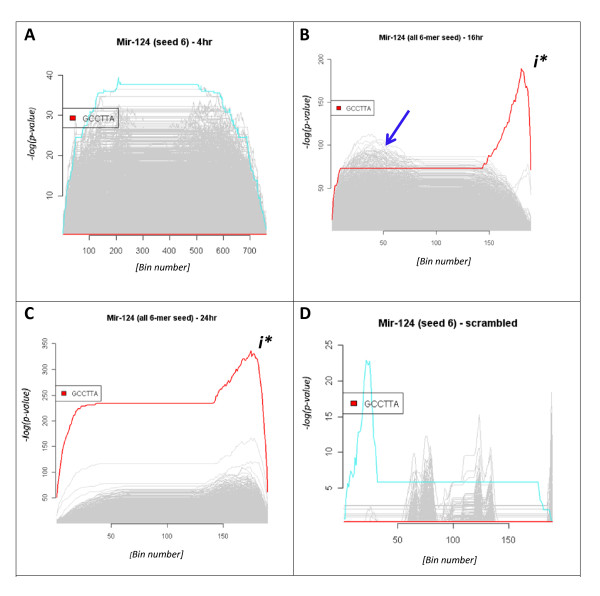
**Composite profile plots**. To contextualise how the top scoring hsa-miR-124 hexameric seed query (*i.e*. GCCTTA) enrichment profile compared to that of all other unique nucleotide hexamers, an equivalent analysis was completed using SBSE with each of 4096 (*i.e*. 4^^6^) unique hexameric seed queries. The resulting information is then intuitively represented by composite plots of the, previously described, enrichment score. The composite plots succinctly summarise the estimated enrichment scoring of all 4096 unique hexameric (seed) queries. Each of the four **PanelsA-D** represent the analysis of a separate time-point following hsa-miR-124 transfection. In each instance the x-axis represents the ranked transcripts (*i.e*. by fold change, from most up-regulated (left) to most down-regulated (right)). The x-axes scale indicates the number of bins used in the analysis (See methods for further detail of bin implementation). The y-axis represents the enrichment score which is scaled dependent on the range of enrichment scores encountered within the dataset. Each of grey lines represents the estimated scoring of a unique hexameric query sequence, while the highest scoring hexamer at each time point is coloured turquoise. The hsa-miR-124 GCCTTA seed motif is coloured red throughout and the maximum enrichment score "i*" is indicated on **PanelsB** and **C**. Furthermore, in **Panels B** and **C** the maximum enrichment score is the query sequence. The selected data clearly summarises how the hsa-miR-124 motif gains in prominence in each of the post-transfection samples and becomes undetectable if the differential expression profile is shuffled (**Panel D**). In particular, note how the overall enrichment score of the datasets fluctuates post-transfection. Initially (**Panel A**) all data forms a homogeneous body with no enrichment score above 40. 16 hours post-transfection (**Panel B**) a large number of up-regulated AT-rich transcripts are obvious (indicated by the blue arrow). After 24 hour post-transfection (**Panel C**) this collection of up-regulated transcripts are no longer apparent.

Analysing the data as described gave enrichment profiles that indicated significant enrichment of the query motif in isolation (*i.e*. a query enrichment score had no context). In an effort to capture how the enrichment scores of specific queries related to that of the potential motif universe (*i.e*. 4 to the power 6 equates to 4096 unique hexamers) of a dataset, each of the differential expression profiles were queried sequentially with all 4096 unique nucleotide hexamers, to assess how specific query motifs were relatively enriched. Our analyses indicated that, with the exception of the 4 hour sample, all profile estimates detected an unambiguous and prominent over-representation of the nucleotide complement of the hsa-miR-124 seed query sequence (Figures 3A, B, C and 3D and Additional File 1 Figure S1). A score was considered significant if it was distinctly larger than the majority of other profile estimates. To once more rule out the possibility that these were random observations inherent in a large population each dataset was shuffled (as previously described) and each query repeated. In every instance this simple shuffling of the data completely abrogated the enrichment trace and further supported our assumption that ranked expression profiles can be used to estimate miRNA target enrichment.

Another feature of the data was that of significant fluctuations in the observed enrichment scores of a large number of AT-rich motifs (indicated with a blue arrow in Figure 3B and also with the 8 hour analysis described by Additional File 1 Figure S2). This enrichment peaked at 8 hours before subsiding with each time point. Efforts to determine enrichment of specific ontological terms were inconclusive (not shown).

The differential expression profiles of each respective time point was queried with a variety of motifs that encompassed the 5' hsa-mir124 seed region. From the resulting plots it was observed that the hexamer GCCTTA generated the maximum enrichment score of 320 and that the 24 hour post-transfection expression profile was the most enriched for the complement of the hsa-miR-124 seed motif (Figure 3C). Equivalent profile plots generated using the heptamer query TGCCTTA also produced a significant enrichment score of 250 (Additional File 1 Figure S1), indicating that nucleotide position 7 may also be a highly conserved and functional residue. The TGCCTT motif generated an enrichment score of 120, suggesting a significant functional role for the adenine residue in hsa-miR-124 RNAi activity (Additional File 1 Figure S1). Comparing the various expression profiles emphasised the transient nature of the RNAi effect and that the narrower enrichment peaks observed at 16 and 24 hour post-transfection suggest these are the optimum time points with which to maximise identification of the direct hsa-miR-124 target transcripts. These combined observations strongly support our view that the enrichment score can be used as a simple measure of hsa-miR-124 RNAi and that the approach enables a simple and rapid evaluation of miRNA seed region conservation.

This dataset was also used to investigate the effect of binning an expression dataset. A wide range of bin sizes (*i.e*. 100-19000) were investigated and in each instance consistency of scores detected was observed irrespective of the bin size used to group the data (Additional File 1 Figure S3) though there are obvious implications regarding computational processing time (*i.e*. calculation times increase with increasing bin sizes).

### Case study 2

Six Affymetrix GeneChip^® ^Human Genome U133 Plus 2.0 cel files were retrieved from database entry E-MEXP-875. This dataset was generated to investigate the effects of FAM33A RNAi knockdown on the gene expression profile in a lung carcinoma cell line[30]. Two unique siRNA oligonucleotides were used in duplicated transfections, one of the sense strands being CGAUUUAAAUAUAUGUACA- dTdT (FAM33A_1) and the other GGCUGGAAUAUGAAAUCAA- dTdT (FAM33A_2). The two remaining samples acting as a non-silencing, control dataset. Our primary hypothesis in this instance was that if biological off-target activity of either siRNA occurred via a miRNA-like transcript down-regulation mechanism then it should be possible to detect enrichment of putative off-target transcripts (*i.e*. using enrichment of the complementarity seed motif as a proxy of transcript down-regulation), as described for the hsa-miR-124 dataset. Furthermore, if the result indicated this to be a valid assumption it should be possible to use the enrichment plots and scores to differentiate the two siRNA with regard to their off-target interference potential (*i.e*. select the siRNA with the smallest off-target interference potential).

**Figure 4:**
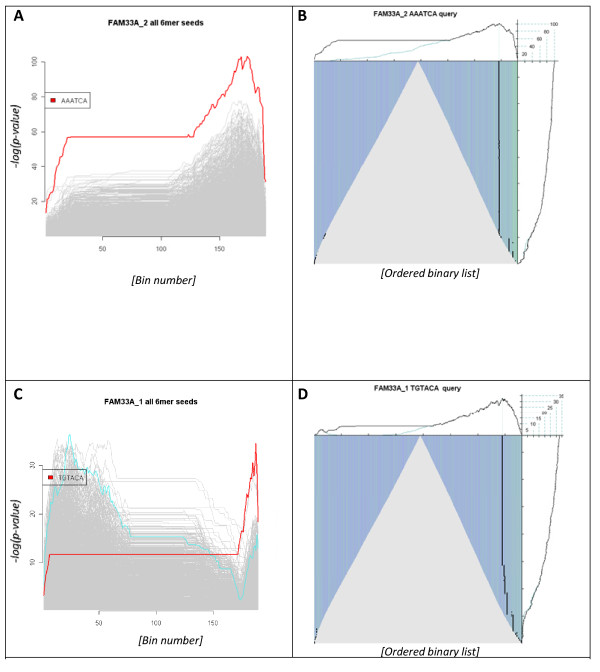
**Comparison of two FAM33A siRNA transfection studies**. The first column (**Panels A** and **C**) represent respective composite plots summarising the enrichment scoring of all possible hexameric queries given each of the FAM33A siRNA differential expression profiles (See panel headers for specifics). As before, the highest scoring hexamer is coloured turquoise while specific query motifs are coloured red. The x-axes again represent the bin number. The second column (**Panels B** and **D**) summarises the analysis of each differential expression profile with the highest scoring motifs, of the two FAM33A siRNAs, both of which were identified using the respective composite plots (*i.e*. A with B and C with D). All plot decorations are consistent with previous descriptions. Note the intense enrichment profile of the FAM33A_2 siRNA (indicated by the green vertical lines) relative to FAM33A_1 estimate. This indicates a more significant off-target 'profile' following transfection with the FAM33A_2 siRNA, relative to that of the FAM33A_1 siRNA. Also note how the enrichment plot scaling differs between the two queries (*i.e*. 0-100 in **Panel B** and 0-35 in **Panel D**)

Composite plots summarising the enrichment scores of all 4096 unique hexamer nucleotide queries indicated that enrichment peaks were associated with the down-regulated transcripts of both siRNA differential expression profiles (Figures 4a and 4C). The largest enrichment score, and most distinct profile, was observed with the FAM33A_2 transfection dataset, with the AAATCA hexamer (Figures 4A and 4B). This motif corresponds with residues 2-7 of the anti-sense strand of the FAM33A_2 siRNA. A much narrower and less prominent peak was observed in the FAM33A_1 transfection dataset with the TGTACA motif (Figures 4C and 4D). This motif corresponds with the 5' anti-sense end of the FAM33A_1 siRNA. These observations suggest that the anti-sense strand of both siRNAs may encode miRNA-like off-target activity.

When each highest scoring hexamer was further investigated it was readily apparent from the respective graphical summaries that the FAM33A_2 *siRNA *AAATCA motif was encoded in the 3'UTRs of a significant number of the most down-regulated transcripts, and in contrast, that the FAM33A_1 siRNA profile was close to that of background, involving few of the most down-regulated transcripts (Figures 4A, B, C and 4D). Further single query analyses involving all possible derivations of motif queries encompassing the respective putative anti-sense seed regions generated less significant scores (not shown). Randomising each dataset (as described above) completely abrogated both of the observed peaks (not shown) again emphasising that the enrichment score is dependent on the ranking of sequence universe and is not an artefact of a large dataset. An additional detail reported in the original publication is that the FAM33A_1 siRNA down-regulated the FAM33A transcript approximately 10-fold while the FAM33A_2 siRNA down-regulated the FAM33A transcript approximately 6-fold. This combined with our reported observations suggest the FAM33A_1 siRNA is a more efficacious agent with a reduced off-target potential relative to the FAM33A_2 siRNA and would be the siRNA of choice for any future applications.

### Case study 3

E-MEXP-456 consists of six Affymetrix GeneChip^® ^Human Genome U133 Plus 2.0 cel files. In this investigation the effect of an siRNA knock-down (*i.e*. an antagomir) of the human miR-30a-3p miRNA precursor was evaluated in HepG2 cells in an attempt to identify hsa-miR-30a-3p target transcripts [31]. One would hypothesis that if the transfected siRNA were to prevent hsa-miR-30a-3p (UGUAAACAUCCUCGACUGGAAG) transcript repression, then the complement of the hsa-miR-30a-3p seed motif should in turn be enriched in the up-regulated transcript population following transfection with the siRNA duplex.

**Figure 5:**
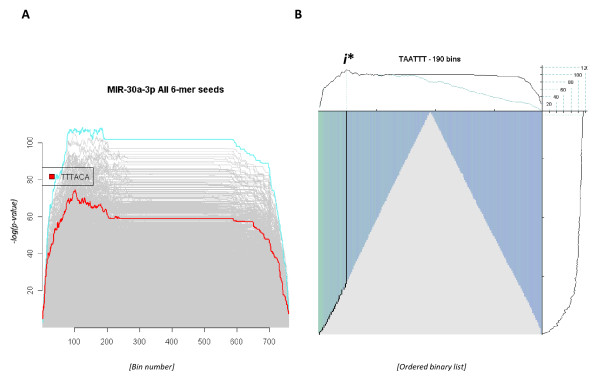
**siRNA knock-down of miR-30a-3p**. (**Panel A**) A composite plot summarising the enrichment scoring of all possible hexameric queries given the associated hsa-miR-30a-3p differential expression profile. The complement of the hsa-miR-30a-3p seed motif (TTTACA) is highlighted in red. All other higher scoring hexamer profiles represent uncharacterised AT-rich hexamers. Also note the enrichment scores of greater than 100 with a number of AT-rich motifs among the up-regulated transcripts located on the left of the plot. **(Panel B) **This plot summarises the enrichment profile of the highest scoring hexamer (highlighted in turquoise in the composite plot of **Panel A**) following hsa-mir-30a-3p transfection. SBSE analysis estimates this motif (TAATTT) to be enriched in the up-regulated transcripts and the maximum enrichment score is therefore located to the left of the plot.

A potential drawback to this dataset is that few of the transcripts are significantly dysregulated at this time point. Hierarchical clustering does not clearly differentiate between control and treatment samples, while a volcano plot reports that only 70 transcripts demonstrate a >1.5-fold change in expression with an associated p-value of <0.05 (Additional File 1 Figure S6). Clearly, detecting enrichment of potential seed motifs given the limited treatment effect requires a sensitive estimating method. That noted, composite plots summarising the enrichment of every possible nucleotide hexamer (Figure 5A) indicated that a number of motifs, including the complement of the hsa-miR-30-3p seed motif (*i.e*. TTTACA), were enriched in the up-regulated transcript population. However, the most significant enrichment scores were associated with a number of AT-rich hexamer motifs (*e.g*. TAATTT, TTTAA and ATATTT). Intriguingly this motif does not represent either the major or minor forms of hsa-miR-30a-3p, but it was noted that a similar composite profile (*i.e*. enrichment for AT-rich hexamers) was observed when analysing the 8 hour time-point of the hsa-miR-124 time-series[7] (Additional File 1 Figure S2A) and in other analyses of RNAi microarrays datasets (not discussed). In the time series analysis the AT-rich enrichment was superseded by enrichment for the complement of the miRNA seed motif at all subsequent (*i.e*. post 8 hours transfection) time points. This emphasised the transitory nature of expression profiling and it is tempting to speculate that this AT-rich feature represents a general cellular response to RNA transfection and that enrichment of the complement of the hsa-miR-30a-3p seed motif would have become more pronounced with time in a manner analogous to that observed with the previously described time-series. However, additional post-transfection data would be required to confirm this hypothesis. As with previous analyses, shuffling the association between fold-change and transcript identifier resulted in no significant peak detection with equivalent motif queries (not shown). This observation adds further evidence to our proposition that the direct effects of miRNA activity can be inferred by enrichment of the complement of miRNA seed motif.

### Case study 4

Six Affymetrix GeneChip^® ^Human Genome U133 Plus 2.0 cel files were retrieved from database entry E-GEOD-16097. This dataset included 3 replicates of a control transfection and 3 replicates of an siRNA transfection designed to knock-down the human BAHD1 transcript in HEK293 cells [32]. Total RNA was extracted 72 hour post-transfection using standard protocols. This dataset was unique in that the treatment samples were transfected with a cocktail of three siRNAs designed to perturb human BAHD1 mRNA transcripts. The sense strands of the siRNAs used in the study were; BAHD1_1 GGUCAAUGGCAAGAACUAU- dTdT, BAHD1_2 GGCUGCCCUGAUGAACCAU- dTdT and BAHD1_3 GGACUUGCAUUUUCAGUUU_ dTdT.

**Figure 6:**
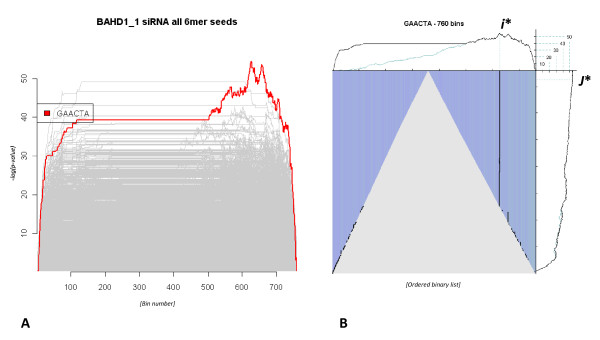
**Knock-down of BAHD1 with an siRNA cocktail**. (**Panel A**) Composite plot summarising the enrichment scoring of all possible hexameric queries given the BAHD1 siRNA transfected differential expression profile (as described in the main text). As before, the query sequence is indicated by a red line and in this instance is also observed as the highest scoring hexamer. The x- and y-axes represent bin number and enrichment score, respectively. The most significant score that can be attributed to any of the siRNA transfection pool is GAACTA which is a complementary match of the 5' end of the negative strand of siRNA BAHD1_1 and indicative of off-target interference by the this strand. **(Panel B) **This plot summarises the enrichment profile of the highest scoring hexamer (as highlighted in red in the composite plot of **Panel A**) within the differential expression profile of the BAHD1 siRNA transfection dataset. SBSE analysis estimates the GAACTA motif to be enriched in the down-regulated and by inference is acting in a miRNA-like repressive manner. Both i* and j* (see methods for detailed definitions of both symbols) are indicated on the upper-most and rightmost plots, respectively.

A composite plot summarising the profile estimates of all 4096 unique nucleotide hexamers found no evidence of significant BAHD1_2 or BAHD1_3 complement siRNA seed motifs using nucleotide queries that encompassed both the sense and anti-sense siRNA strands (not shown). However, a modest but most significant enrichment score was observed with the BAHD1_1 siRNA motif GAACTA (Figure 6) that indicated potential off-target signalling dictated by the 5' end of the negative siRNA strand. As with earlier analyses this signal was abrogated when the expression profile was 'randomised'. By inference we propose that this signal is indicative of a miRNA-like off-target effect unique to the BAHD1_1 siRNA and that if all else were equal either the BAHD1_2 or BAHD1_3 siRNAs should be used in further RNAi transfections in preference to the BAHD1_1 siRNA.

### Case study 5

**Figure 7:**
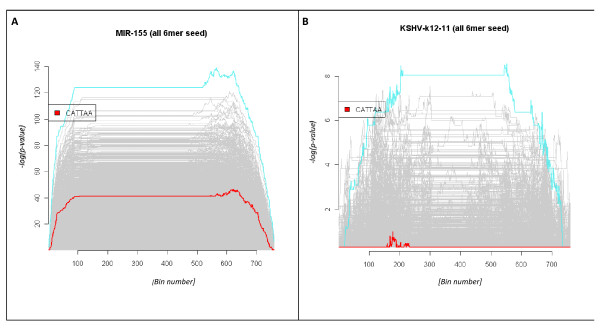
**Comparing miRNA orthologues**. Composite plots summarising the enrichment scoring of all possible hexameric queries given the respective hsa-miR-155 (**Panel A**) and KSHV-miR-k12-11 (**Panel B**) differential expression profiles. In each instance the highest scoring hexamer is highlighted in turquoise while the highest scoring seed hexamer is highlighted in red. Note how the hsa-miR-155 profile is dominated by AT-rich transcripts and that there is minimal enrichment of the miRNA target motif. The paucity of significant motifs in the KSHV-miR-k12-11 plot highlight that few transcripts are differentially expressed relative to the control dataset. Also note that the seed region is conserved in both miRNAs.

Previous case studies indicated that our Bayesian estimate scores in combination with composite profiles can be used to differentiate siRNA of the same target specificity by comparison of their respective predicted off-target profiles. An extension of this observation is that transfections comparing reported miRNA orthologues should, in principle, produce similar differential expression profiles (*i.e*. each relative to a negative control) if the respective miRNA target the same gene transcripts. The E-GEOD-9264 dataset was considered ideal to test this assumption as it consisted of 12 Affymetrix GeneChip^® ^Human Genome U133 Plus 2.0 cel files, four of which were control replicates (pCDNA3.1), four transfected with hsa-miR-155 and four samples transfected with the KSHV-miR-k12-11 miRNA, a proposed orthologue of hsa-miR-155 [33]. Overlapping nucleotide motif queries of the hsa-miR-155 seed region (UUAAUGCUAAUCGUGAUAGGGGU) indicated a modest enrichment score for the 6-mer CATTAA that was distributed across approximately one third of the down-regulated 3'UTR space (Additional File 1 Figure S9C). The corresponding composite graph (Figure 7A) is similar to that observed with the Wang[7] 8 hour post-transfection observation (Additional File 1 Figure S2A) in that the predicted complement of the miRNA seed motif although enriched is not amongst the most significantly enriched hexamers. Considering our observations with the hsa-miR-124 time study data we propose that the hsa-miR-155 enrichment profile also varies with time and that the observed profile in this instance may not indicate the maximum enrichment potential (and by inference, inhibitory profile) of this miRNA. It was also noted that a GCATTA query (*i.e*. the complement of hsa-miR-155 residues 2-7) resulted in a minimal enrichment score of 30, indicating that the first residue position is highly conserved in the target transcript 3'UTRs (not shown). As with previous datasets shuffling the transcript dataset abrogated this signal (not shown).

When equivalent motif queries derived from the KSHV-miR-k12-11 seed region (UUAAUGCUUAGCCUGUGUCCGA) were used to query the equivalent KSHV-miR-k12-11 transfected dataset no significant peaks could be detected (Figure 7B). This observation suggested that the KSHV-miR-k12-11 seed region motif is not a major determinant of the observed differential expression profile. Hierarchical clustering and heat map representations of the most differentially expressed transcripts (Additional File 1 Figure S9) suggest that the KSHV-miR-k12-11 transfected dataset is more similar to the control data than the equivalent hsa-miR-155 dataset. Given that both miRNA transfections were equivalent in every other respect the combined observations suggest that it may be premature to describe hsa-miR-155 and KSHV-miR-k12-11 as miRNA orthologues.

## Discussion

The key assumption implemented within SBSE is that both 'direct' miRNA down-regulatory events and siRNA off-target interference can be accurately assessed via estimates of 'seed' motif enrichment in a ranked sequence population. Enrichment estimates are calculated using common microarray summary statistics and a weighted Bayesian analysis of the ranked sequence space. Each estimate is presented as a simple, but intuitive graphical summary to facilitate an understanding of how the RNAi event under investigation may have dictated the observed differential expression profile. The approach is particularly attractive in that it requires minimal assumptions about either the method of inhibition, or the characteristics of the transcript targets (*i.e*. transcript interference requires the presence of a complementary seed sequence motif and enrichment of this motif is indicative of RNAi activity). Given that a single miRNA is capable of down-regulating multiple transcripts [15,34,35] we reasoned that combined these simple assumptions could be used to calculate an estimate of 'direct' miRNA target via enrichment of the respective miRNA seed target motif. Furthermore, widespread siRNA off-target transcript inhibition has been reported to be mediated via a miRNA-like seed region complementarity [21-23]. By extension, equivalent estimates may be used to assess and compare miRNA-like off-target inhibition. Our approach is similar to the Sylamer algorithm [29] which estimates for "word" enrichment in a given ranked gene list using a cumulative hypergeometric distribution function. However, SBSE may improve on such estimates by using microarray summary statistics to direct a sequential data-driven analysis of the data that preferentially 'weights' for the most significant changes in expression. Weighting the data in this way appears to increase sensitivity of the enrichment estimate and has enabled us to apply our method to estimate weaker enrichment profiles, for example, those associated with siRNA off-target transcript inhibition. We are confident that these estimates are indicative of RNAi directed inhibition and not the result of some unforeseen structure inherent in a large dataset, as a simple randomisation of the sequence-fold change relationship abrogated significant estimates in every instance.

Our analyses indicate that a SBSE approach can be used to infer the optimum timing, magnitude and likely location of 'direct' RNAi events. Analysis of a hsa-miR-124 post-transfection time-series [7] emphasised the transient nature of miRNA induced changes of the global expression profile and underlines the risk associated with generating hypotheses and validation studies based on a single, arbitrary, post-transfection sample. That is, a differential expression profile derived from a sub-optimal time-point may actually represent a 'generic' cellular perturbation response rather than specific RNAi. In several instances we observed enrichment of AT-rich motifs that appear to represent cellular responses to 'foreign' RNA[34], as they fluctuate with time, and conclude that such datasets are likely to be misleading if they are extrapolated to infer direct RNAi effects. That said analyses of several fixed time-point case-study datasets were included to investigate the utility of our estimates under diverse experimental conditions. Our analysis found that reported miRNA orthologues did not result in similar perturbations of the global expression profile. This suggests that the miRNA in question should not be annotated as orthologues until further validation studies have been completed. By iteratively querying each respective dataset with over-lapping nucleotide seed region queries we were able to generate hypotheses regarding the degree of seed region conservation (*i.e*. elevated enrichment scores equate with increased conservation of that motif in the target transcript 3'UTRs). Our analyses are in agreement with previous observations; of a conserved adenine anchor [16] as higher estimate scores were generally observed with query motifs that included an "A" as a first residue; and of minimal sequence conservation immediately downstream of the seed region. The latter observation emphasises the need to further define these apparently un-conserved determinants of target specificity [35,36] if we are to extend our knowledge beyond that of the critical seed region.

Of particular interest to our group is the ability to better understand and minimise siRNA off-target effects, as such events may either limit the utility of a siRNA being used as a therapeutic agent or compromise interpretation of functional knock-down studies. Current opinion is that such undesired responses are the result of innate immune responses [37] and miRNA-like transcript inhibition [21-23]. The former are generally attenuated by chemical modifications of the siRNA olignonucleotides [38] though the method of deliver may also generate unwanted cellular effects [34]. Given available data our analyses suggest that siRNA miRNA-like effects are a magnitude less than that observed with similar miRNA transfection studies. This is to be expected given that siRNA miRNA-like off-target activity is a chance event and in contrast to the conserved and concerted miRNA signalling networks that dictate cellular differentiation [39]. Furthermore, each respective siRNA off-target profile appears to be a sequence, and strand, specific characteristic. Our observation is in agreement with previous studies that reported siRNA off-target events to be both sequence and species-specific [40,23]. This is a significant conclusion as these simple estimates can be used to differentiate equally efficacious siRNA molecules based on their off-target potential. Such observations will have obvious application both in the development of siRNAs as therapeutic agents and molecular functional tools.

## Conclusion

Common microarray summary statistics combined with a simple Bayesian analysis have proven sufficient to estimate the magnitude of the direct RNAi transcription effect. Our analyses indicate that SBSE can be used to infer the optimum timing, magnitude and likely location of 'direct' RNAi events, and is sufficiently sensitive to differentiate siRNAs of similar efficacy but with different off-target signalling potential.

## Methods

The SBSE algorithm was implemented as an R script http://www.r-project.org/ and is free to download for use and further evaluation (See Additional File 4). All data and supporting data files required to replicate the reported results have also been included as additional information (Additional File 3). Development and testing was completed using version R-2.9.1 on both Red Hat^® ^Linux and Microsoft Windows XP.

### Concept

Assume that as part of a miRNA functional characterisation effort we have determined the, relative to control, fold-changes of six gene transcripts. These respective expression values are used to generate a simple ordered (*i.e*. from most up-regulated to most down-regulated) list. We hypothesis that if the observed differential expression profile has been caused by the transfected miRNA, the down-regulated transcripts (*i.e*. those to the right) will be enriched with target sites for the miRNA seed region relative to that observed with the up-regulated transcripts (*i.e*. those to the left). Let's suppose that our list is populated with the following expression values [4, 2, 0.5,-0.2,-1.5,-3]. Each of the six transcript identifiers is then associated with its respective 3'UTR sequence via a one-to-one mapping matrix, and using a simple pattern matching function we determine the presence or absence of a specified nucleotide hexamer, in each of the respective 3'UTR sequences. The pattern matching results are used to transform our ordered list into an 'ordered' binary list indicating a match, or no match, of the nucleotide query (seed) sequence in each of the respective 3'UTR sequences of our ordered list. Assume that this takes the form [0, 0, 1, 1, 0, 1].

A second list is derived from the ordered fold-change data, but in this instance the data indicates the absolute ranking of each differentially expressed transcript and for our illustration takes the form [1,6,5,4]. The absolute ranking is used to direct a step-wise analysis of the differential distribution of 1's as they are encountered in the binary vector. That is, for each increment of the absolute list we calculate the lowest likelihood that the binary profile observed to-date can be best explained by the differential distribution of 1's between the leftmost and rightmost transcripts. For the simple case described we begin with an up-regulated gene not containing a seed sequence match and hypothesise that there is a one-half chance that the differential expression profile observed at this point is due to differences in miRNA distribution. Next, we observe a down-regulated gene containing a seed sequence match fall to the right, and update our hypothesis accordingly. We next observe another up-regulated gene not containing a seed sequence match *etc*. The algorithm continues to update the hypotheses until all of the data has been evaluated. On completion we are left with five values (*i.e*. one for each hypothesis) corresponding to each division between the six observations. This analysis procedure is represented in cartoon format in Figure 1A.

On completion of the analysis the estimated probabilities are plotted alongside a simple graphical representation (See Figure 1B) that summarises how the algorithm navigated the dataset and the estimated likelihood at each interval. These combined observations are used to determine the maximum enrichment score of the query (seed) motif in the dataset and, by inference, to quantify the likely magnitude of the miRNA repression of gene transcription given the observed fold-change dataset. Using our simplified example each row of the main plot corresponds to an additional observation. The vertical black lines indicate the optimal division as the data is processed. The black line of the uppermost plot (U) summarises the -log of the estimated p-value for each division and is used to determine the optimal (lowest) value (i*). The blue line summarises a *post hoc *calculation of the p-value for each division when the number of observations is optimal (j*). The black line of rightmost plot (R) also summarises the -log of the estimated p-value associated with each hypothesis update and is used to determine optimal division (j*) of the dataset (*i.e*. the number of observations needed to estimate the lowest p-value). In our illustrative example we propose that only the four most differentially expressed observations are required to estimate the optimum partitioning of the data (*i.e*. the most likely location of miRNA repression). The blue line summarises a *post hoc *calculation to estimate the p-value using the optimal partitioning of the dataset.

The following assumptions are made with regard to the dataset: (1) that the 3'UTRs represented the full length transcript, and (2) that only one query (seed) match per 3'UTR was of relevance to the transcript repression mechanism (3) that RNAi transcript targets are down-regulated post-transfection.

### Algorithm details

Let "D" denote our ordered binary list of length N (the total number of gene transcripts). In this list a one corresponds to the *i*^th ^gene's UTR having a seed sequence match, while a zero corresponds to the *i*^th ^gene's UTR not having a seed sequence match. Now let "A" denote our absolute data list, also of length N. Initially consider the top j transcripts of list A. This value is used to extract and partition the *j*-most differentially expressed transcripts from D.

Now let *H_i, j _*denote our hypothesis that the differential distribution of 1's observed between the division D_1...j _(the "left") and D_j+1...N _(the "right") can be best explained by the distribution of the miRNA seed motif on either side of this division. Let  denote the number of ones in the left set and  the number of zeros in the left set. Similarly,  is used to denote the number of ones in the righst set, and  the number of zeros in the right set.

Given the above definitions we define an updating mechanism that allows the complete dataset to be traversed and our hypothesis to be incrementally evaluated. First, an initial estimate is assigned to the hypothesis *H*_*i*,*j*-1_, that is, the probability that our hypothesis is correct given that we have observed D_*j*-1 _(*i.e*. *j*-

1) transcripts. This probability is updated for each subsequent A[*j*] increment of the dataset and can be succinctly defined using the Bayes' formula.(1.1)

Note that P{H*_0_*|D_*1*...*j*-1_} = 1- P{H_*i*, *j*-1_|D_*1*...*j*-1_} is the probability that the differential expression at this division cannot be explained by the observed distribution of the miRNA seed motif (*i.e*. the seed motif distribution is random).

Should the next most differentially expressed transcript of encode a miRNA seed motif then a logical assumption is that the probability of the next transcript falling to the left of the division is the current ratio of seed sequences matches to the left of the division to the total number of seed sequences matches.(1.2)

Further, the probability of the next transcript falling to the right of the division is the current ratio of seed sequences matches to the right of the division to the total number of seed sequences matches observed.(1.3)

However, if the next transcript does not have a seed sequence match, then the probability of the next transcript falling to the left of the division is the current ratio of transcripts without a seed sequence match to the left of the division to the total number of transcripts without a seed sequence match observed.(1.4)

By extension, the probability of this transcript falling to the right of the division is the current ratio of transcripts without a seed sequence match to the right of the division to the total number of transcripts without a seed sequence match observed.(1.5)

Under our null hypothesis all of the observed differential expression is assumed to be independent of the miRNA seed motif distribution. Hence the probability that the next transcript falls to the left of the division is the ratio of transcripts to the left of the division to the total number of transcripts.(1.6)

Likewise, under the null hypothesis the probability that the next transcript falls to the right of the division is quite simply the ratio of transcripts to the right of the division to the total number of transcripts.(1.7)

The P{H_*i, 0*_} is given an initial value of 0.5 and equation 1.1 updated until the dataset has been traversed. On completion the highest value of  corresponds to the optimum partitioning of the data. This will be referred to as the optimum enrichment score henceforth and is the most likely estimate that the observed differential expression profile is best explained in terms of the miRNA seed motif distribution by dividing the top j_* _genes at division i_*_. The estimated probabilities, P{H_*i, j*_}, for each D[*j*] are plotted as black lines as indicated in Figure 1B. The estimates  and  are plotted as blue lines in the uppermost (U) and rightmost (R) plots of the summary plots but are not utilised further in this report

### Miniscule values

Our Bayes formula while mathematically correct, may prove problematic as both P{H*_i, j_*|D_1..*j*_} and P{H*_0_*|D_1...*j*_} become miniscule for large datasets. It is therefore pragmatic to work on a logarithmic scale, that is:(2.1)

And likewise,(2.2)

### Binning

The algorithm as defined requires N(N-1) iterations of formula 1.1 to complete an analysis. Given that a typical microarrays dataset summarises the expression data of several thousand genes, it is a valuable option that the dimension of the dataset be reduced to enable a more rapid execution of the analysis. One proven approach is to group the ordered gene list into M bins and apply equation seven for M(M-1) iterations. Under this scenario most of the utilised formulae remain unmodified. However, the functions used to estimate P{D_1...*j *_|H_*i, j*-1_} and P{D_1...*j*_| H_*0*_} must be updated to accommodate this additional option. This can be achieved as follows. Let *x *denote the number of genes with a seed sequence match and *y *the number of genes without a seed sequence match in bin D*_j_*. Under the hypothesis H_*i, j*-1_, the probability that we will observe *x *genes with a seed sequence match falling to the left of the division, and *y *genes without a seed sequence match falling to the left of the division is P{D_1...*j *_|H_*i;j*-1_} = p^*x*^q^*y*^, where(3.1)

If the bin falls to the right of the division, then P{D_1...*j *_|H_*i;j*-1_} = p*^x^*q*^y^*, where(3.2)

Under the null hypothesis P{D_1...*j *_|H_0_} = p^*x+y*^, where(3.3)

should the bin fall to the left of the division and(3.4)

should the bins fall to the right of the division.

### Composite hexamer plot

The summary plot of the query (seed) distribution (See Figure 1B) is a useful representation of the differential distribution of a query sequence and, by inference, an estimate of the magnitude and location of transcript repression in a given dataset. However, an obvious extension of such an estimate is to compare the distribution of a specific query motif relative to that of all other possible query sequences of the same length (*i.e*. to evaluate our specific seed query estimate in context with all other putative explanatory seed sequences). To address this requirement the SBSE algorithm was extended to iteratively query a given dataset with a comprehensive library of 4096 (*i.e*. 4^^6^) unique hexamer nucleotides and plot each of the resulting estimates on a composite graphical representation. Such plots allow a simple and succinct graphical representation of how our estimate of a given hexameric nucleotide query motif compares relative to all other hexameric sequences (see Figure 3).

### Datasets

To develop and validate SBSE public microarrays datasets were retrieved from the EBI's ArrayExpress [41] public archive http://www.ebi.ac.uk/arrayexpress. For each study relevant cel files were quality assessed using standard metrics and subsequent expression values RMA normalised [42] before differential expression profiles were generated using the LIMMA library [43]. Human 3'-UTRs were retrieved from BioMart [44] and mapped to Affymetrix probeset identifiers. The longest 3'-UTR was selected when many-to-one UTR mappings occurred. Complex nucleotide repeat patterns were masked using DUST [45].

Brief summaries of selected case studies used in the development and evaluation of SBSE are as follows:

(1) The E-GEOD-6207 dataset is comprised of 14 Affymetrix GeneChip^® ^Human Genome U133A Plus 2.0 cel files. In this study hsa-miR-124 was over expressed in HepG cells and RNAs extracted at time points 0, 4, 8, 16, 24, 32, 72 and 120 h post-transfection [7]. This time course dataset was used extensively to develop several aspects of the SBSE algorithm

(2) Six Affymetrix GeneChip^® ^Human Genome U133 Plus 2.0 cel files were retrieved from E-MEXP-875. This dataset was originally generated to investigate the effects of FAM33A RNAi knockdown on the gene expression profile of a lung carcinoma cell line [30]. Two unique siRNA oligonucleotides were used in separate transfections along with a non-silencing oligonucleotides control.

(3) The E-MEXP-456 dataset consists of six Affymetrix GeneChip^® ^Human Genome U133 Plus 2.0 cel files. In this investigation the effect of siRNA duplex knock-down of the human miR-30a-3p miRNA precursor was evaluated in HepG2 cells in an attempt to identify hsa-miR-30a-3p target transcripts [31].

(4) The dataset E-GEOD-16097 is comprised of six Human Genome U133Plus 2.0 cel files [32]. Briefly, the author used a cocktail of three siRNAs to knockdown the BAHD1 transcript. In each instance HEK293 cells were transfected with either BAHD1 siRNA or control siRNA. Total RNA from cells transfected for 72 h were extracted and purified before hybridization on GeneChip Human Genome U133Plus 2.0 chips.

(5) The E-GEOD-9264 dataset is comprised of 12 Affymetrix GeneChip^® ^Human Genome U133 Plus 2.0 cel files. Four of these were control replicates (pCDNA3.1), four samples transfected with hsa-miR-155 and four samples transfected with the KSHV-miR-K12-11 miRNA, a proposed ortholog of hsa-miR-155 [33].

## Abbreviations

(RNAi): RNA interfering; (miRNA): microRNA; (siRNA): small interfering RNA; (SBSE ): Simple Bayesian Seed Estimate; (3' UTR): 3' untranslated region

## Competing interests

The authors declare that they have no competing interests.

## Authors' contributions

PW conceived the project. MP implemented the algorithm. MP and PW performed the analyses. PW wrote the paper. All authors read and approved the final manuscript.

## Supplementary Material

Additional file 1 **Additional supporting SBSE plots and comparative Sylamer plots**. Click here for file

Additional file 2 **Parsed and masked 3'UTR human sequences necessary to complete a SBSE estimate**. Click here for file

Additional file 3 **Processed Affymetrix datasets described in this report**. Click here for file

Additional file 4 **SBSE R scripts and README.txt required to execute a SBSE estimate**. Click here for file
