# Supplementary material for: A simple Bayesian estimate of direct RNAi gene regulation events from differential gene expression profiles
Source: BMC Genomics. 2011 May 20;12:250. doi: 10.1186/1471-2164-12-250 (PMC3128064; doi:10.1186/1471-2164-12-250)
Supplement: Additional file 1 — Additional supporting SBSE plots and comparative Sylamer plots. [file 1471-2164-12-250-S1.PDF]

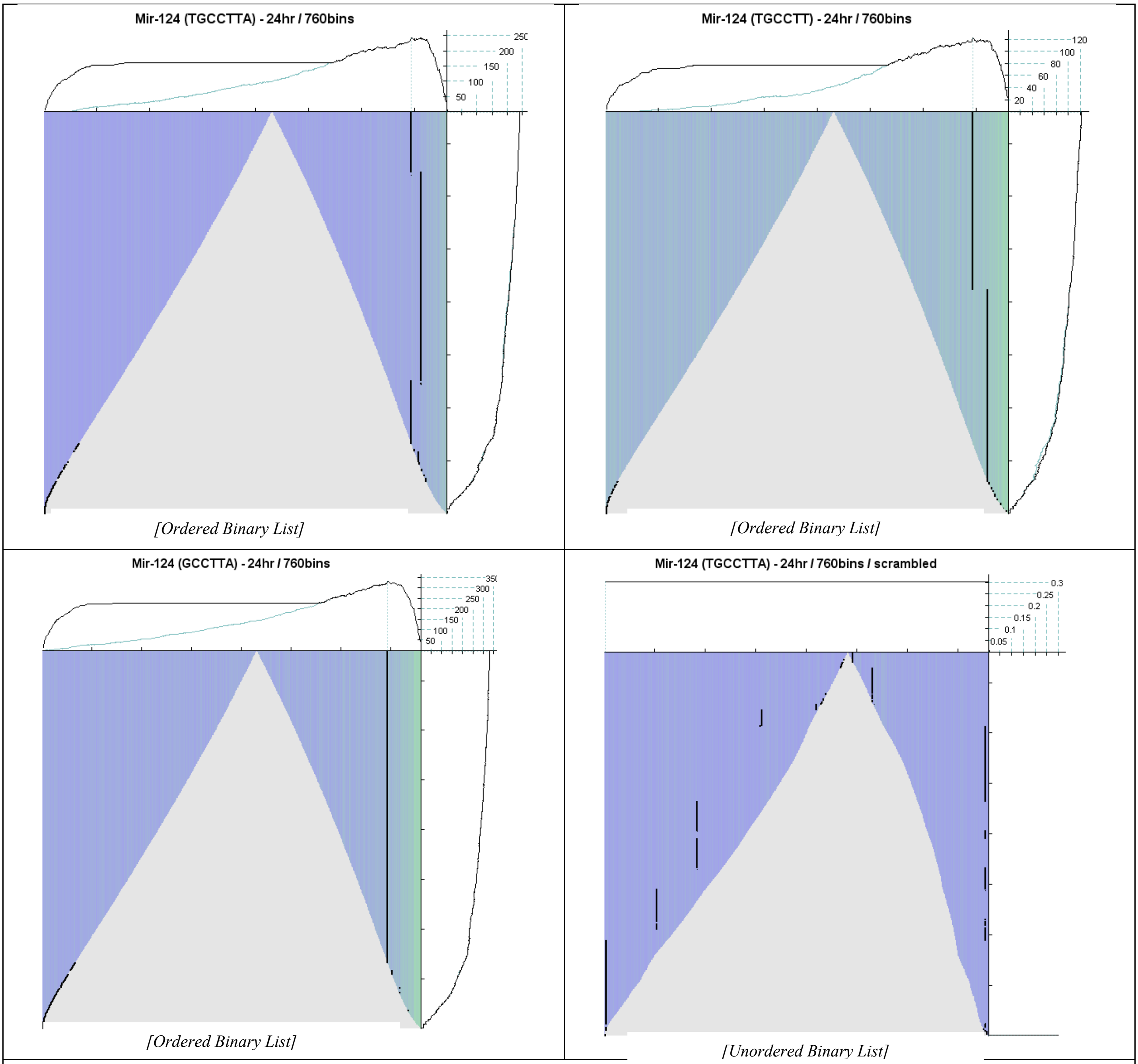

Figure S1: SBSE plots of hsa-miR-124 seed queries. For each plot the x-axes represents our observed fold change data ranked from most up-regulated to most down-regulated. As described in the methods section, the central body of the plot is a graphical summary detailing how the algorithm traversed the dataset. The characteristic 'triangle' emphasises the broadly 'normal' distribution of the dataset and that each dataset is evaluated sequentially from the outermost, and most dysregulated transcripts, toward the central transcripts that were not differentially expressed following RNAi transfection. The green vertical lines represent those 3'UTRs with a perfect match to the query motif while blue vertical lines indicate no query match. The thick black line indicates the optimal division given the data observed to-date. The uppermost plot of each of the pour panels describes the enrichment score and also indicates the location of the most significant partitioning of the data with a vertical dashed line. The rightmost plot of each of the four panels also describes the enrichment score but details the enrichment score for each hypothesis update. **From top left and then clockwise** – a heptamer query encompassing n1-n7 of the hsa-miR-124 seed region equates to a maximum enrichment score of 250. A hexamer query of n2-n7 equates to score of 120, while the hexamer equating to n1-n6 equates to a score of 320. The final plot (bottom right) indicates that no significant score when the dataset is shuffled and supports our proposal that the enrichment score can be used as a proxy estimate of RNAi activity.

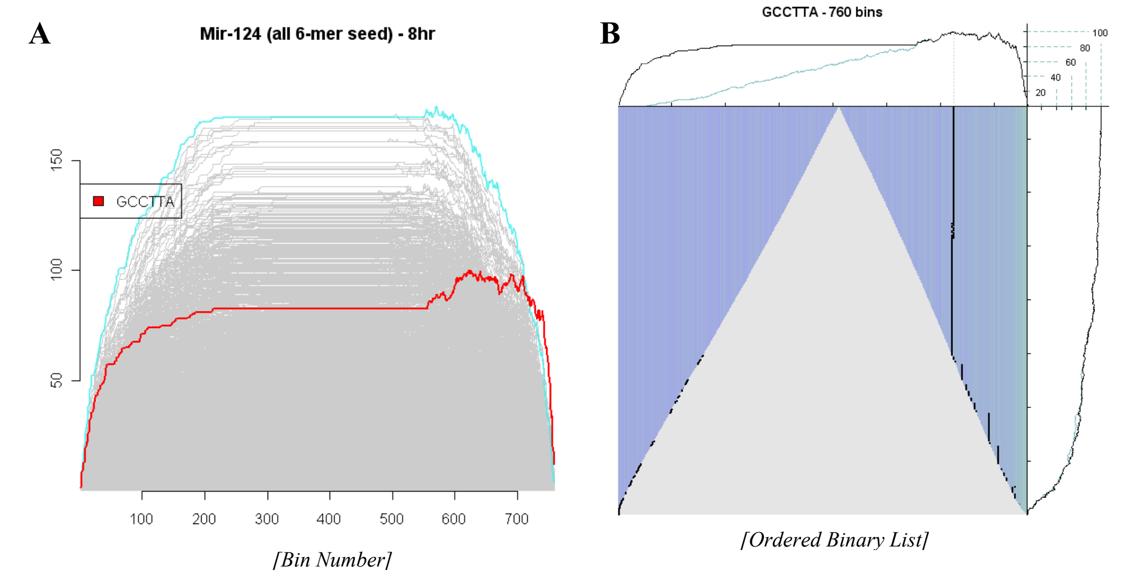

Figure S2: hsa-miR-124 analysis of the 8hour post-transfection sample. The composite plot on the left **(A)** highlights the progressive enrichment of the hsa-miR-124 seed motif GCCTTA as time increases post-transfection (*i.e.* the query sequence score has increased relative to that observed with the 4 hour profile). Note that the most significant enrichment scores are seen with AT-rich nucleotide motifs and are associated with the up-regulated transcripts. This observation is discussed further In the main text. As described previously the red line indicates the enrichment score of the query motif while the turquoise line indicates the enrichment score of the highest scoring nucleotide query. The x-axes describe the number of bins used in the estimation process.

The plot on the right **(B)** is of the enrichment observed with the GCCTTA motif using the 8 hour differential expression profile. The combined plots indicate 'preliminary' enrichment of the motif of the query motif in the down-regulated transcripts though considerably dispersed relative to that observed at later time points. Note that the uppermost plot summarising the enrichment score is the equivalent information to that summarised by the red enrichment line in the composite plot on the left.

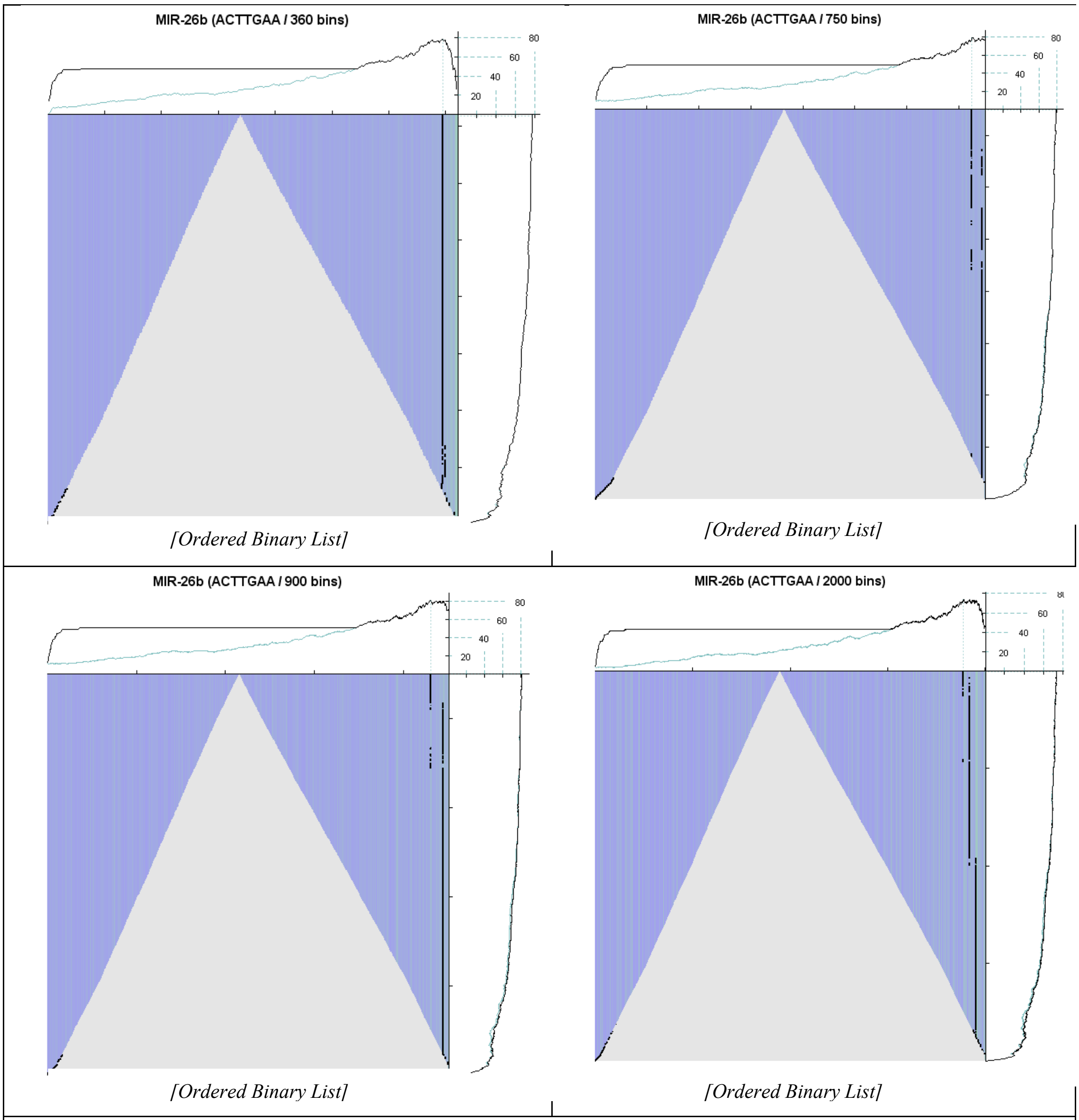

Figure S3: Given that a typical differential expression profile comprises several thousand transcripts and that a single estimate is calculated from N(N-1) iterations of the main SBSE function, where N is the number of probe identifiers, the calculation time quickly becomes problematic. To improve computational efficiency we evaluated the effect of binning transcript data to reduce the dimension of the dataset to M(M-1), where M represents the bin size (See methods for details of bin implementation). Of several bin sizes evaluated there were minimal observed differences in the estimated maximum enrichment score. The hsa-miR-26b transfection data used in this particular investigation was derived from the microarray data made public by Gennarino *et. al.* (2009). As in previous analysis representations the x-axes represents the binary list of the differential expression data arranged from most up-regulated (left) to most down-regulated (right), while the upper and lower graphical summaries of the 4 panels plot the enrichment score (See the methods section for further details).

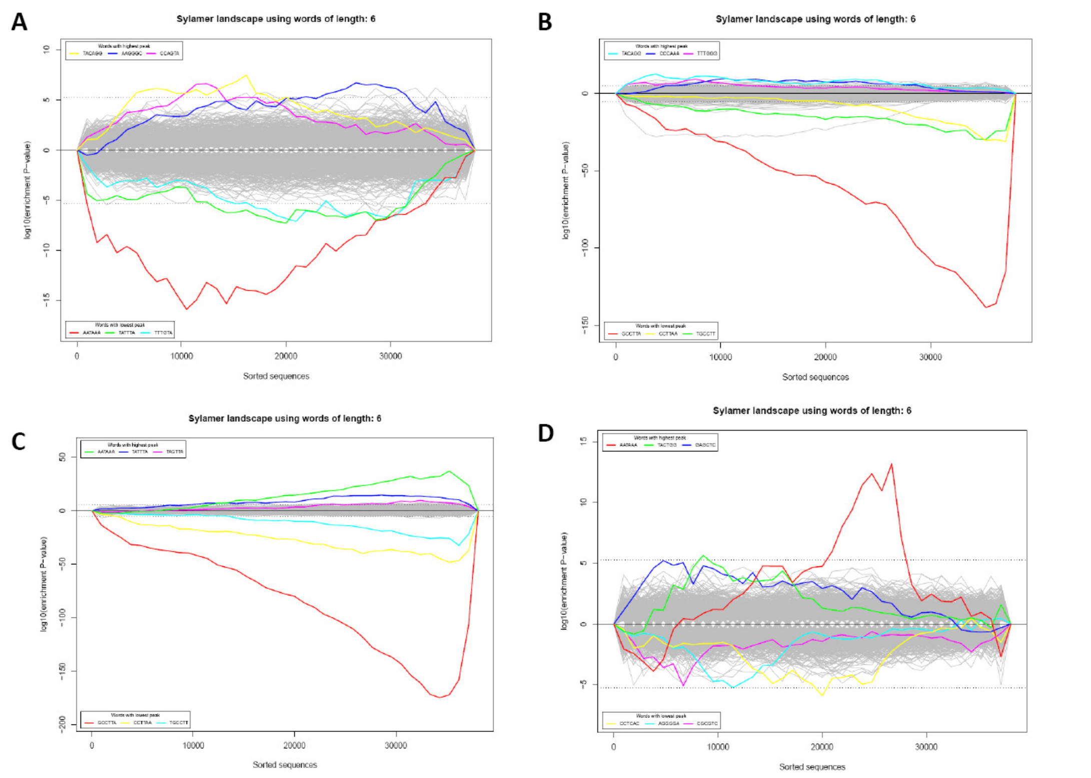

Figure S4: This collection of plots was generated using the Sylamer[(33)] application and are the equivalent of those generated using the SBSE algorithm and presented in Figure 3 of the main text. Briefly, the plots (A-D) represent the 4 hour, 16 hour, 24 hour and shuffled (24hr) differential expression profiles of the hsa-miR-124 transfection, respectively. Each plot is in general agreement to that observed with SBSE. That is: the 4 hour time point indicates no enrichment of the query motif but does highlight enrichment of AT-rich transcripts within the up-regulated transcripts (*i.e.* to the lower left of plot A). Both the 6 and 8 hour time points indicate strong evidence of enrichment of the hsamiR-124 recognition motif (i.e. GCCTTA). The shuffled 24 hour data (plot D) abrogates this signal.

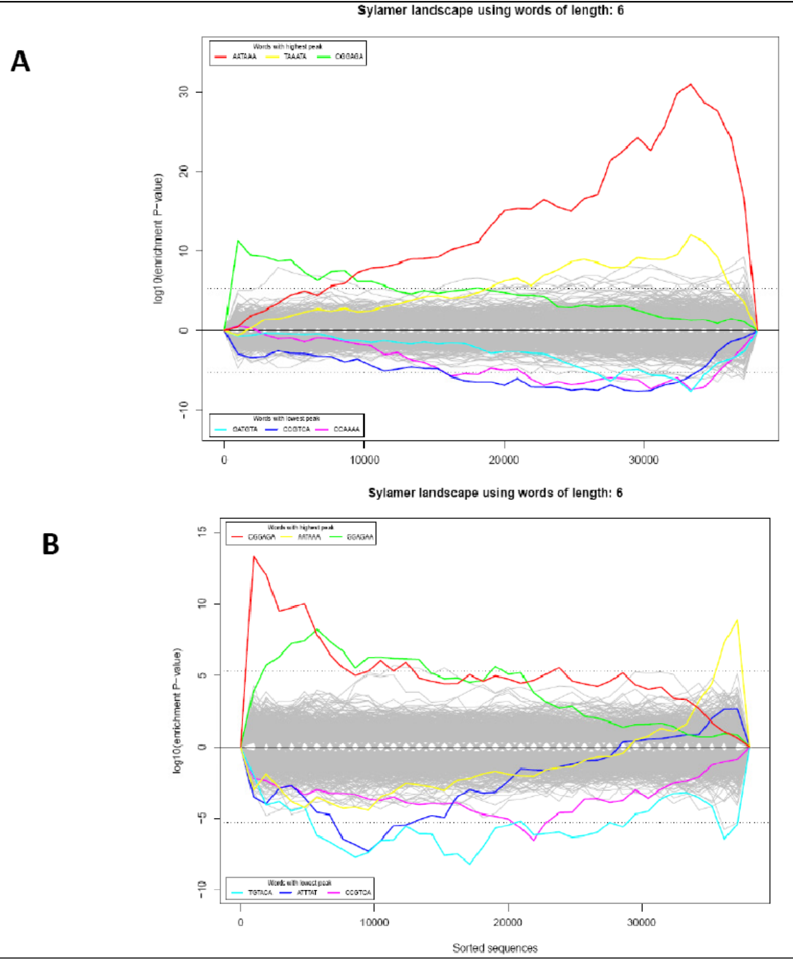

Figure S5: Plots A and B are the equivalent Sylamer analysis plots to that described for SBSE in Figure 4 of the main text. Briefly, plot A was generated using the FAM33A_2 differential expression profile (see methods section for a full description of this dataset). Recall that SBSE identified an AAATCA motif as the most enriched hexamer and that we proposed that this motif was indicative of miRNA-like activity of the 5'-end of the negative strand of the transfected siRNA. Using the same data Sylamer identifies an AATAAA motif as most enriched. This motif is not present in either strand of the transfected siRNA. Plot B was generated using the FAM33A_1 differential expression profile. Using this data SBSE identified enrichment of an ATGTAC motif and we proposed that this was the target motif of the 5'end of the transfected siRNA reverse strand. Sylamer did not report enrichment of any motif in this instance.

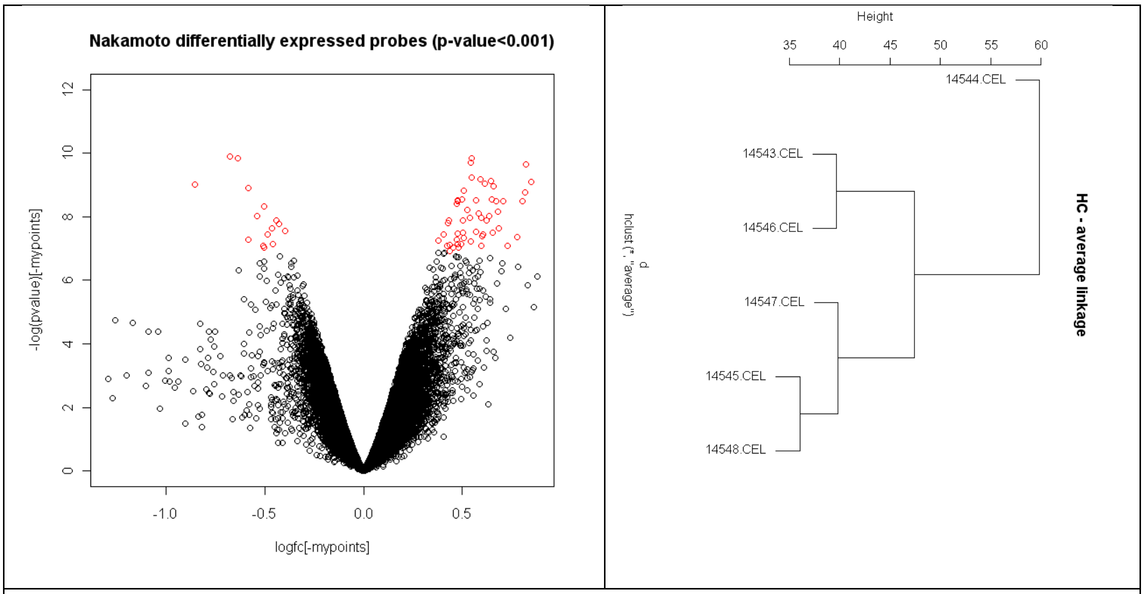

Figure S6: Quality control evaluation summaries of the Nakamoto (2005) RNAi dataset. **(Left)** The x-axes of the volcano plot represents log2 of the fold ratio and indicates that very few probes have a >2 fold change. **(Right)** The hierarchical cluster indicates that control and treated samples do not partition by experimental description: Nodes labelled 14543, 14544 and 14545 represent control transfections 14546, 14547 and 14548 mir30a transfections.

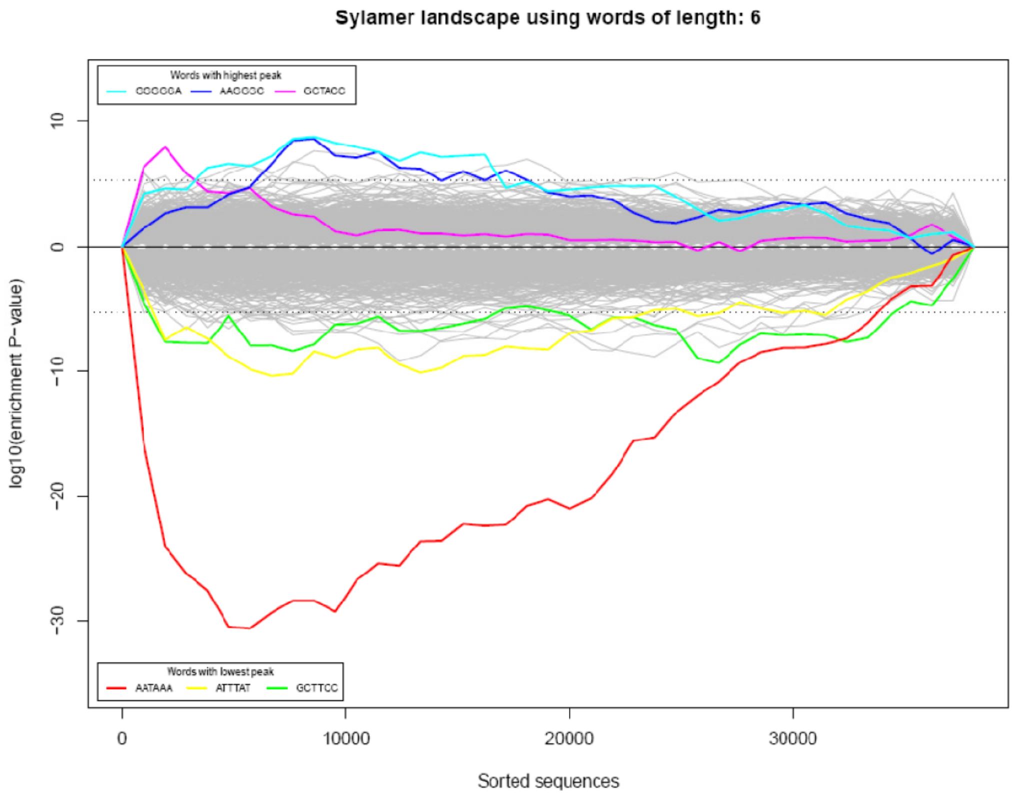

Figure S7: This plot is the Sylamer equivalent of Figure 5A in the main text. Using SBSE we identified modest enrichment of a motif (*i.e.* TTTACA) that we propose was the target site of hsamiR-30a-3p. Enrichment of this motif is not apparent in the equivalent Sylamer plot. Both algorithms identify up-regulated AT-rich motifs as the most enriched motifs in this dataset.

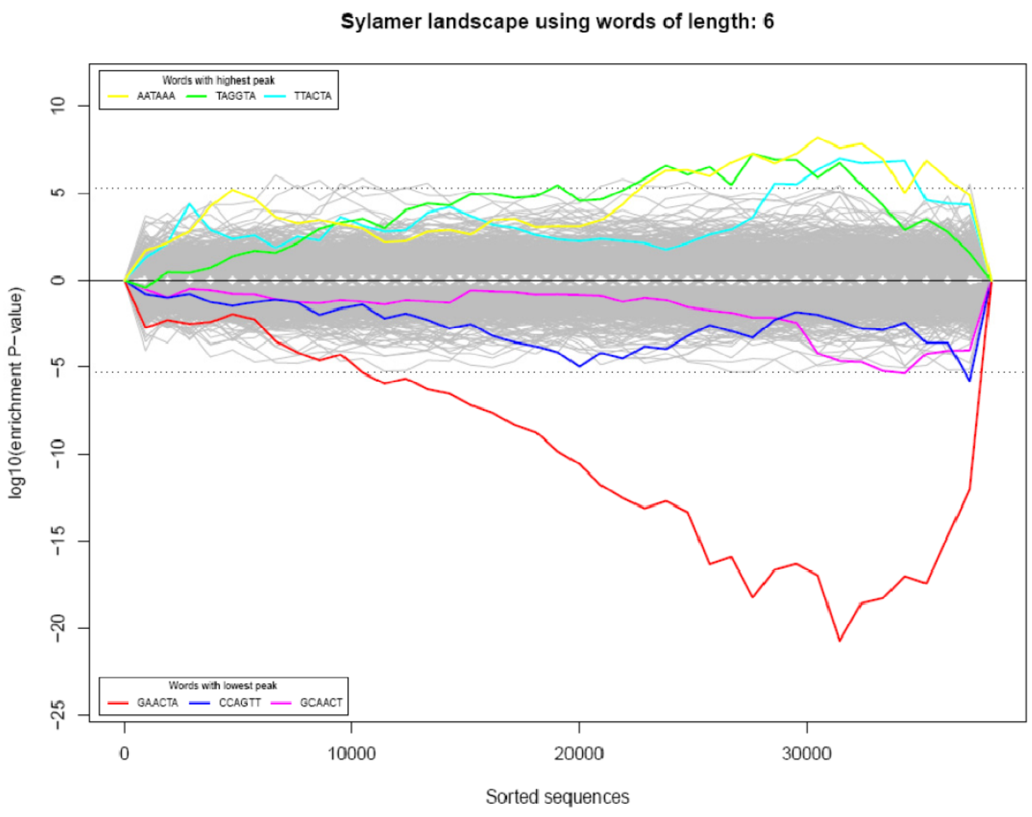

Figure S8: This Sylamer result is the equivalent of the SBSE plot reproduced as Figure 6A in the main text. Sylamer identifies the GAACTA motif as most enriched in the down-regulated transcripts. SBSE also identified this motif as the most enriched within this dataset. We propose that this represents RNAi activity of the transfected BAHD1 siRNA (as described in further detail in the main text).

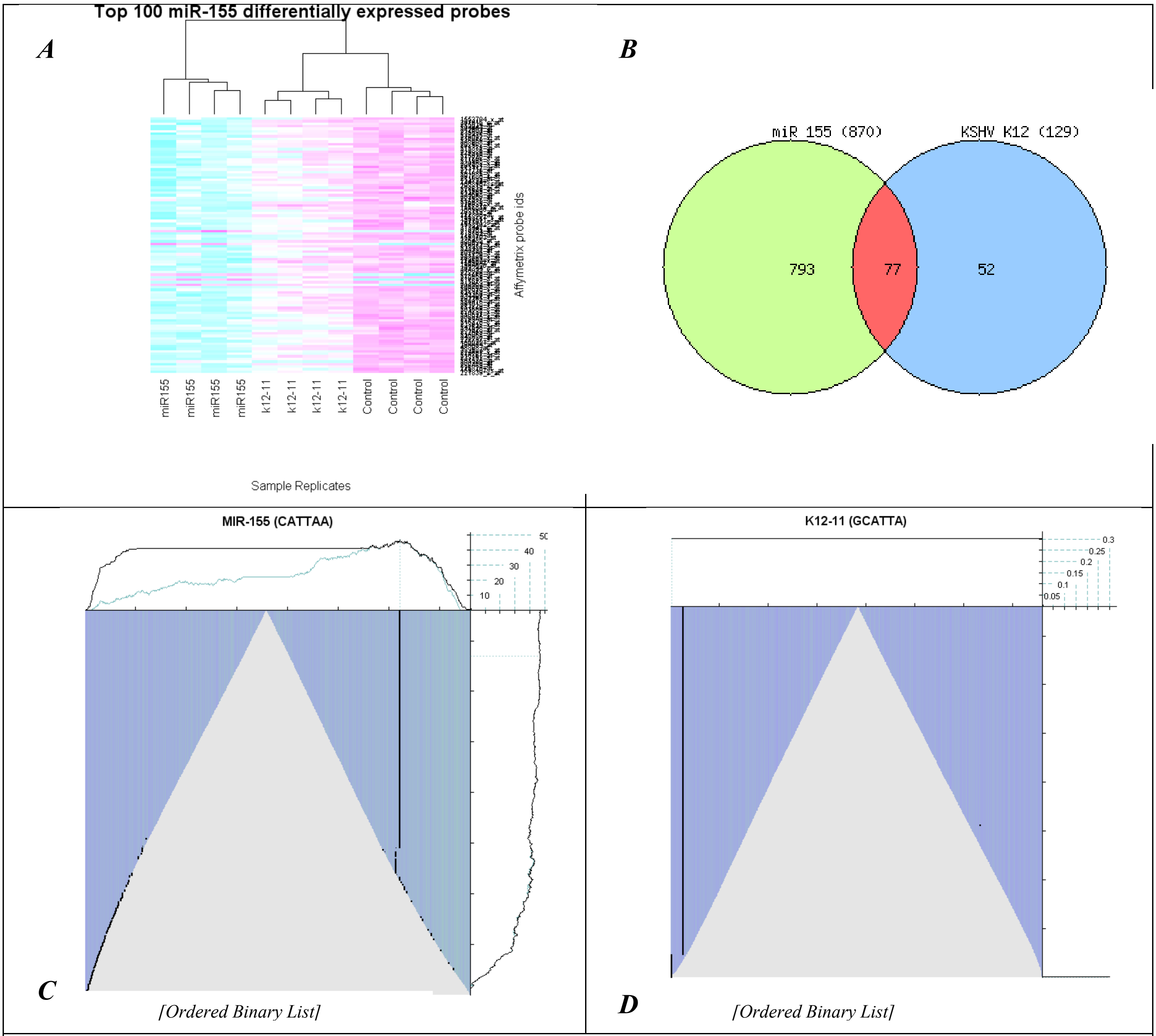

Figure S9: Additional analyses of the Skalsky (2007) dataset. **(A)** Heatmap comparing the 100 most differentially regulated (relative to control) Affymetrix probes following hsa-miR-155 transfection. The map indicates that the KSHV_k12-11 expression profile is more similar to the control replicates than the hsa-miR-155. **(B)** Venn diagram summarising distribution of significantly differentially regulated (FC 1.5> OR <-1.5 and p-value < 0.05 relative to control replicates) Affymetrix probes following transfection with either hsa-miR-155 or KSHV-miR-k12-11. This analysis supports the previous observation that KSHV_k12-11 replicates are more similar to the control samples than to the hsa-miR-155 replicates. **(C)** SBSE analysis of the hsa-miR-155 5′ seed sequence shows a modest enrichment of the query motif. **(D)** An equivalent SBSE analysis of the KSHV-miR-k12-11 5′ seed motif fails to detect any enrichment of the query motif.

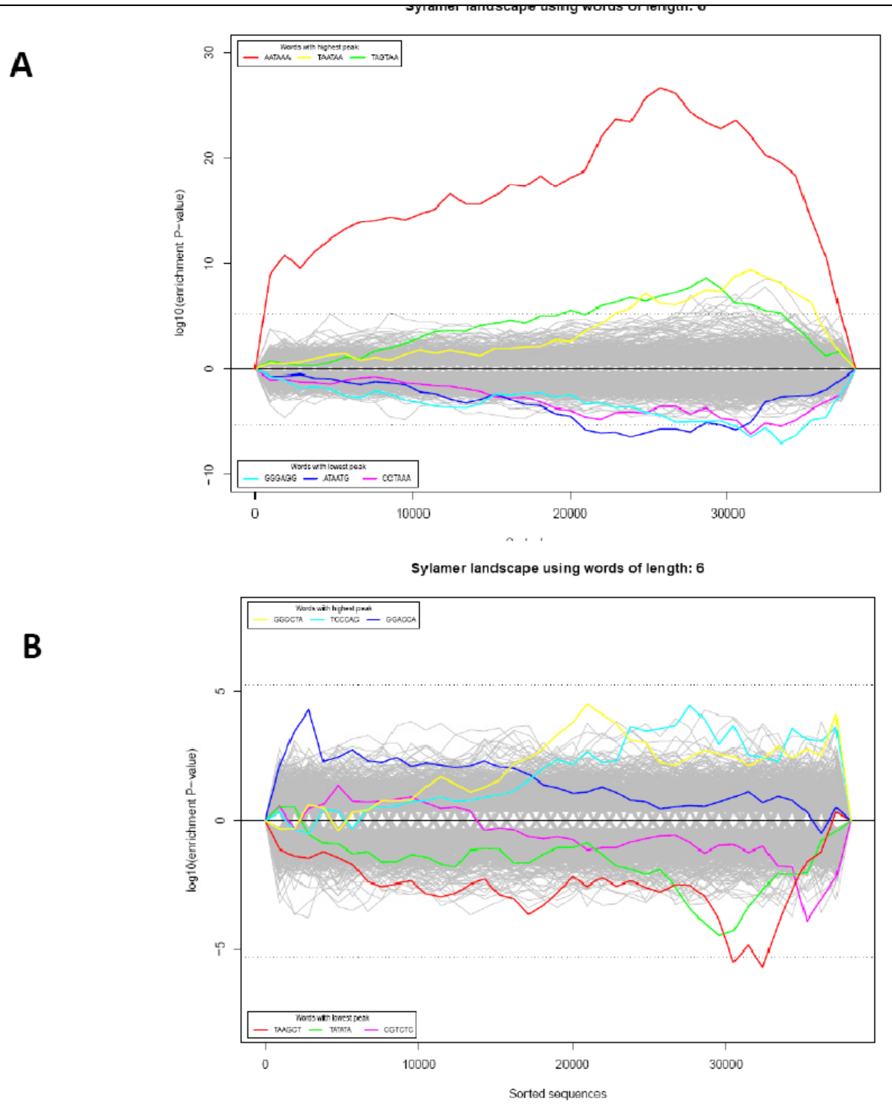

Figure S10: Plot A and B are the Sylamer equivalent of the SBSE plots summarised in Figure 7 of the main text. Neither Sylamer nor SBSE identified the hsa-miR-155 or KSHV-miR-k12-11 seed motif as most significantly enriched in the respective datasets. We propose that the transfection was not optimised and that the resulting differential expression profiles could be used to determine the optimum window for investigating miRNA activity.

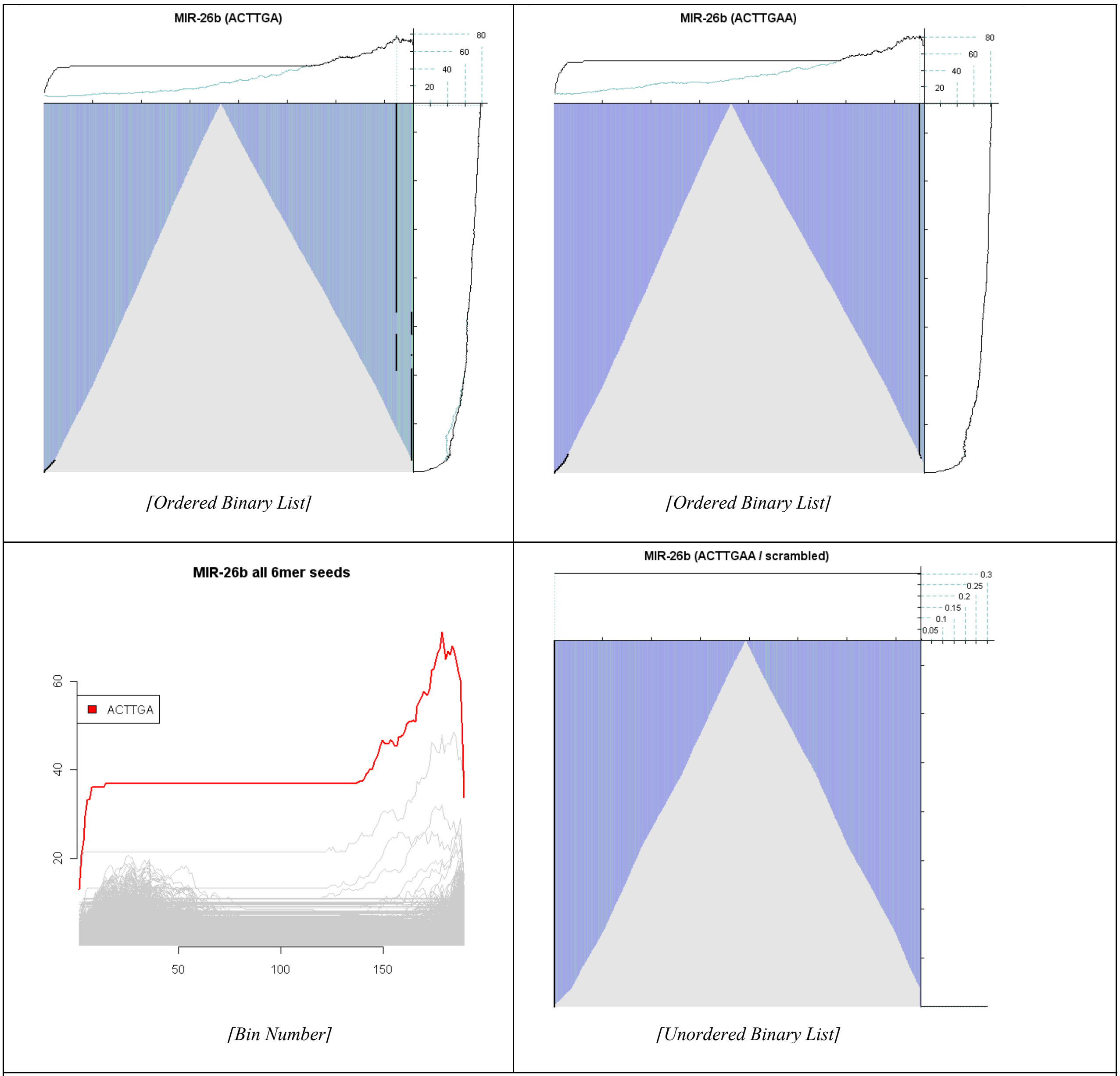

Figure S11: SBSE analyses of the hsa-miR-26b transfected data as reported by Gennarino (2009). The combined plots indicate significant enrichment of the hsa-miR-26B seed region motif and that this enrichment is abrogated when the expression data is shuffled.

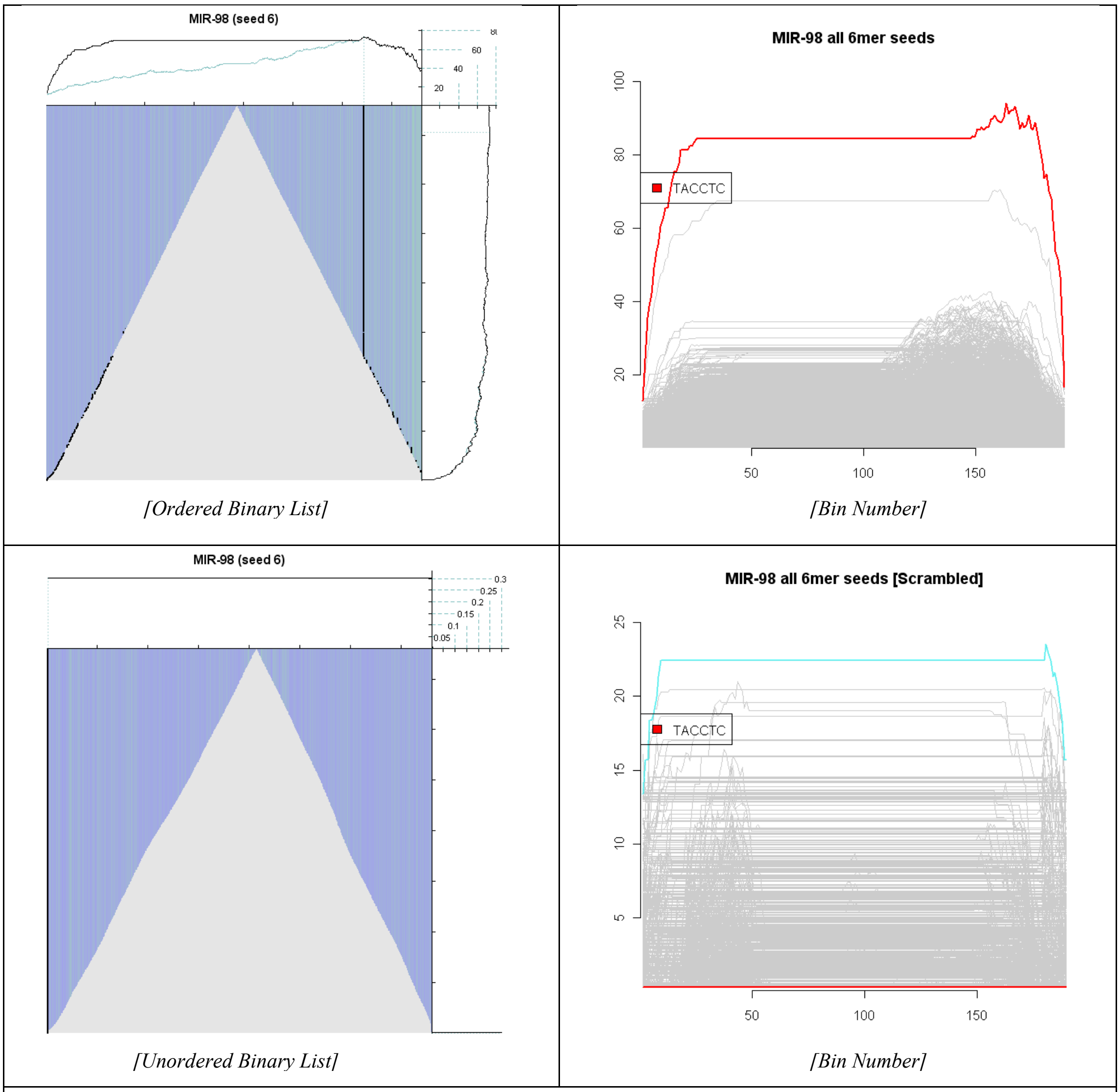

Figure S12: This dataset was dataset was generated using a hsa-miR-98 transfection and made public by the same author as above. Again the combined observations indicate specific enrichment of the 5’ seed motif in the down-regulated mRNA transcripts and abrogation of the enrichment peak when the expression profile is ‘shuffled’. These latter two examples along with a number of other miRNA datasets suggest SBSE has general applicability in the detection of RNAi directed perturbations of transcript expression.
